# Classification of paediatric brain tumours by diffusion weighted imaging and machine learning

**DOI:** 10.1038/s41598-021-82214-3

**Published:** 2021-02-04

**Authors:** Jan Novak, Niloufar Zarinabad, Heather Rose, Theodoros Arvanitis, Lesley MacPherson, Benjamin Pinkey, Adam Oates, Patrick Hales, Richard Grundy, Dorothee Auer, Daniel Rodriguez Gutierrez, Tim Jaspan, Shivaram Avula, Laurence Abernethy, Ramneek Kaur, Darren Hargrave, Dipayan Mitra, Simon Bailey, Nigel Davies, Christopher Clark, Andrew Peet

**Affiliations:** 1 Institute of Cancer and Genomic Sciences, School of Medical and Dental Sciences, University of Birmingham, Birmingham, UK; 2 Oncology, Birmingham Women’s and Children’s NHS Foundation Trust, Birmingham, UK; 3 Department of Psychology, School of Life and Health Sciences, Aston University, Birmingham, UK; 4 Aston Neuroscience Institute, School of Life and Health Sciences, Aston University, Birmingham, UK; 5 Institute of Digital Healthcare, WMG, University of Warwick, Coventry, UK; 6 Radiology, Birmingham Women’s and Children’s NHS Foundation Trust, Birmingham, UK; 7 Developmental Imaging & Biophysics Section, UCL Great Ormond Street Institute of Child Health, London, WC1N 1EH UK; 8 The Children’s Brain Tumour Research Centre, University of Nottingham, Nottingham, UK; 9 Sir Peter Mansfield Imaging Centre, University of Nottingham Biomedical Research Centre, Nottingham, UK; 10 NIHR Nottingham Biomedical Research Centre, Nottingham, UK; 11 Medical Physics, Nottingham University Hospital, Queen’s Medical Centre, Nottingham, UK; 12 Neuroradiology, Nottingham University Hospital, Queen’s Medical Centre, Nottingham, UK; 13 Department of Radiology, Alder Hey Children’s Hospital NHS Foundation Trust, Liverpool, UK; 14 Haematology and Oncology Department, Great Ormond Street Children’s Hospital, London, UK; 15 The Newcastle Upon Tyne Hospitals NHS Foundation Trust, Newcastle, UK; 16 Sir James Spence Institute of Child Health, Royal Victoria Infirmary, Newcastle upon Tyne, UK; 17 Radiation Protection Services, University Hospitals Birmingham NHS Foundation Trust, Birmingham, UK

**Keywords:** Medical research, Oncology

## Abstract

To determine if apparent diffusion coefficients (ADC) can discriminate between posterior fossa brain tumours on a multicentre basis. A total of 124 paediatric patients with posterior fossa tumours (including 55 Medulloblastomas, 36 Pilocytic Astrocytomas and 26 Ependymomas) were scanned using diffusion weighted imaging across 12 different hospitals using a total of 18 different scanners. Apparent diffusion coefficient maps were produced and histogram data was extracted from tumour regions of interest. Total histograms and histogram metrics (mean, variance, skew, kurtosis and 10th, 20th and 50th quantiles) were used as data input for classifiers with accuracy determined by tenfold cross validation. Mean ADC values from the tumour regions of interest differed between tumour types, (ANOVA *P* < 0.001). A cut off value for mean ADC between Ependymomas and Medulloblastomas was found to be of 0.984 × 10^−3^ mm^2^ s^−1^ with sensitivity 80.8% and specificity 80.0%. Overall classification for the ADC histogram metrics were 85% using Naïve Bayes and 84% for Random Forest classifiers. The most commonly occurring posterior fossa paediatric brain tumours can be classified using Apparent Diffusion Coefficient histogram values to a high accuracy on a multicentre basis.

## Background

Brain tumours are the most common solid tumours in childhood and the largest cause of death from cancer in this age group. About half of the tumours arise from the posterior fossa with the most common site being the cerebellum making them amenable to surgical resection but with a significant risk of subsequent morbidity. The degree of the resection required is dependent on the type of tumour and so pre-operative diagnosis is desirable as it can aid in surgical planning. The patients have magnetic resonance imaging at presentation as a standard of care giving the opportunity to achieve this.

Discrimination of the three main types of brain tumours in the posterior fossa (Ependymoma, Medulloblastoma and Pilocytic Astrocytoma) using qualitative assessment of MRI is challenging due to overlapping radiological characteristics but can be improved by the inclusion of diffusion weighted imaging (DWI)^1–3^. However, there is increasing evidence that identification of tumours is improved by using quantitative image analysis and the combination of this with pattern recognition techniques. This has been applied in a single centre study to good effect^4^. Texture analysis of conventional MRI has been implemented in single and multi-centre studies^5–7^ and been applied to DWI in a single centre study^8^. Advanced imaging techniques^9^ such as spectroscopy have shown an ability to differentiate between posterior fossa tumours^10–12^, however the technique is still very challenging to implement in the clinical context due to the reliance on scientific input, inhomogeneous protocols across scanner vendors and a lack for consensus analysis pipelines. Perfusion imaging likewise has acquisition and analysis issues outstanding which make it challenging to implement clinically in routine practice. Texture analysis of T2-weighted MR images, has been shown to discriminate well between tumour types in both single^6,8^ and multi-centre studies^5^.

One imaging modality that has made its way into routine imaging protocols is diffusion-weighted imaging. The apparent diffusion coefficient (ADC) maps are generally produced by scanner software and therefore readily available on clinical PACS systems. ADC is a quantitative measure so does not require normalisation to healthy-appearing tissue. Importantly, we have previously shown ADC values to be reproducible between different scanners and field strengths using standard clinical protocols (including scanners used in this study), which is essential for effective multicentre studies^13^.

ADC values have previously been shown to discriminate between posterior fossa paediatric brain tumours but for Medulloblastomas and Ependymomas, where significant overlap between ADC values is observed for the two tumour types. Some studies have found significant differences^8,14,15^ but others have not^4,10^. One limitation of previous studies and studies of paediatric brain tumours in general are the very small cohort sizes due to the rarity of the diseases. To implement large scale studies of paediatric cancer it is essential to conduct these on a multicentre basis where robust imaging biomarkers will be required^16^.

In this study we present a multicentre analysis of ADC maps focussing on paediatric brain tumours of the posterior fossa. The study involves one hundred and seventeen patients from five primary treatment centres across the UK with scans from twelve hospitals using eighteen different scanners. ADC has been shown to be reproducible across multiple centres and scanners and this large pediatric cohort is a test of the robustness of this approach in a clinical scenario.

## Methods

This study was approved by the Derby Regional Ethics committee and informed consent was obtained from all parents/legal guardians. The consent included the upload of clinical and imaging data to the UK Children’s Cancer and Leukaemia Group Functional Imaging Database. All methods were performed in accordance with the relevant guidelines and regulations.

Whilst each centre was able to set an MRI protocol for patients with new brain tumours, national guidelines exist and are compatible with those adopted in Europe for clinical trials. The protocol includes T1w, T2w and T1w post contrast imaging as well as DWI.

Five primary treatment centres provided data for the study Nottingham, Newcastle, Great Ormond Street Children’s Hospital London, Alder Hey Liverpool and Birmingham Children’s Hospital. Including local hospitals where the children originally presented, MRI were performed at twelve different hospitals on a total of eighteen different scanners in the study. The specifics of the scanners and parameters used for the various scans are summarised in the supplementary material. Histological diagnoses were acquired as per standard clinical practice.

## Image analysis

All images were checked prior to analysis for significant artefacts and image warp sometimes associated with diffusion-weighted imaging. ADC maps were produced using in-house software written in the Python program language using standard, well documented methods. All maps were produced using b = 0 and b = 1000 s/mm^2^ images.

Regions of interest were drawn manually for the whole tumours excluding areas of large cysts and peri-tumoural oedema using MRIcro (version 1.40, http://people.cas.sc.edu/rorden/mricro/). The ROIs were drawn on the b0 images (essentially a T2-weighted image) whilst viewing the complete image set of higher resolution T2/ FLAIR images acquired and using them to determine the margins of the tumour.

The regions of interest were drawn by an expert scientist with six years of experience in neuroimaging with a special interest in paediatric neuroimaging (JN). For inter-user validation of the ROIs, a sub section of the data was semi-randomly selected (8 Medulloblastomas, 6 Ependymomas and 6 Pilocytic Astrocytomas) for re-drawing separately by two radiologists(BP (4 years experience) and AO (13 years experience)). Each radiologist drew ROIs for ten of the patients (20 ROIs in total) and the ADC histogram values were extracted from the tumours. Values from the radiologist’s ROI were then compared against those produced by JN.

ADC values were extracted from the tumour ROIs and placed into 180 bins for histogram analysis using in house software written in Python 2.7. The bin width was 0.022 × 10^−3^ mm^2^ s^−1^. For the histogram analysis and illustrative purposes the data was normalised to the area under the histogram. Post-normalisation, mean histograms were produced by summing the normalised frequency value for each bin separately and dividing by the number of cases. The error bars in the average histogram plots represent one standard deviation for each bin of the normalised frequency.

## Statistics and classification

A receiver operator characteristics curve (ROC) was produced for the ADC mean values within the Medulloblastoma and Ependymoma regions of interest using SPSS (version 22 IBM). The selection of the mean in lieu of the median was arbitrary as no difference was observed in the classification results between the two measures. The box and whisker plot was produced using the R statistical package (The R Foundation, version 0.5.0, 2013). The three main types of paediatric posterior fossa brain tumours were classified with two classification methods, Naïve Bayes (NB) and Random Forest using the Orange Data Mining Tool (Orange, version 2.7.8)^17^.

In detail, the three-way classification method was as follows: The raw histogram data was not used for classification, only extracted values from the histograms (Min, Max, Mean, Median, Variance, Skew, Kurtosis and the 5th, 10th, 20th, 25th, 35th, 40th, 45th, 50th, 55th, 60th, 70th, 75th, 80th, 85th and 90th quantiles) were used as the input data. A principal component analysis was conducted on the input data prior to the cross validation for data reduction covering 95% of the variance (a maximum of 10 components were used) and the resultant components were classified using NB and RF. The data was validated using 10 folds cross validation.

## Patients

The study included 55 patients with Medulloblastomas, 36 with Pilocytic Astrocytomas and 26 with Ependymomas, 4 Atypical Teratoid Rhabdoid Tumours (ATRTs) and 3 other low grade tumours all found in the posterior fossa. The rarer tumour types (ATRTs and low grade tumours) were included for illustrative purposes but due to small numbers were not included in the classification analysis. All of the various histopathological subtypes within these 3 tumour subgroups were grouped together for purposes of analysis. Patient details are shown in Table 1 including age, the distribution of the field strengths at which the patients were scanned and the mean and the range of the ADC values within the tumour ROIs. 4 posterior fossa ATRTs were included for visual comparison only but were not included in the classification analysis as the numbers were too small. Likewise, for the low grade tumours these were presented visually in the supplementary material and not included in the classification due to the very small numbers (n = 3).

**Table 1:** Some details of the patients included in the study including age, and histogram parameters for the three main tumour types.

Tumour Type	Pilocytic Astrocytoma	Ependymoma	Medulloblastoma
Number of Patients	36	26	55
Field Strength Scanned at: 1.5 T/3 T	30/6	21/5	46/9
Age /years	7.1 ± 4.4	4.7 ± 5.0	6.3 ± 3.5
Age Range	1.2–13.8	1.0–16.3	1.5–14.5
Mean ADC (10^−3^ mm^2^ s^−1^)	1.656 ± 0.290	1.126 ± 0.155	0.870 ± 0.154
ADC Variance	9.49 × 10–8	7.89 × 10–8	1.00 × 10–7
ADC Skew	0.70 ± 0.83	1.34 ± 1.13	1.40 ± 0.60
ADC Kurtosis	3.08 ± 3.50	5.95 ± 10.09	3.81 ± 3.24
10th quantile ADC (10^−3^ mm^2^ s^−^1)	1.345 ± 0.266	0.869 ± 0.128	0.587 ± 0.109
25th quantile ADC (10^−3^ mm^2^ s^−1^)	1.480 ± 0.261	0.970 ± 0.135	0.681 ± 0.138
50th quantile ADC (10^−3^ mm^2^ s^−1^)	1.628 ± 0.291	1.084 ± 0.151	0.806 ± 0.138
75th quantile ADC (10^−3^ mm^2^ s^−1^)	1.799 ± 0.343	1.229 ± 0.171	0.989 ± 195
ADC range (10^−3^ mm^2^ s^−1^)	1.222–2.240	0.865–1.400	0.523 ± 1.230

## Results

The ROIs drawn by the radiologists were compared using the extracted mean ADC values with those from JN. A correlation coefficient of R = 0.977 was observed indicating very good agreement between raters (a Bland–Altman plot for the mean values is included in the supplementary material with a repeatability coefficient of CR = 1.06 × 10^−4^). All of the metrics used for the classification were assessed between the raters and no significant differences were detected between metrics derived from the raters’ ROIs (two-tailed t-test, Bonferroni-corrected for multiple comparisons). This indicated that all of the metrics were reliable and henceforth used for the machine learning classification.

**Figure 1:**
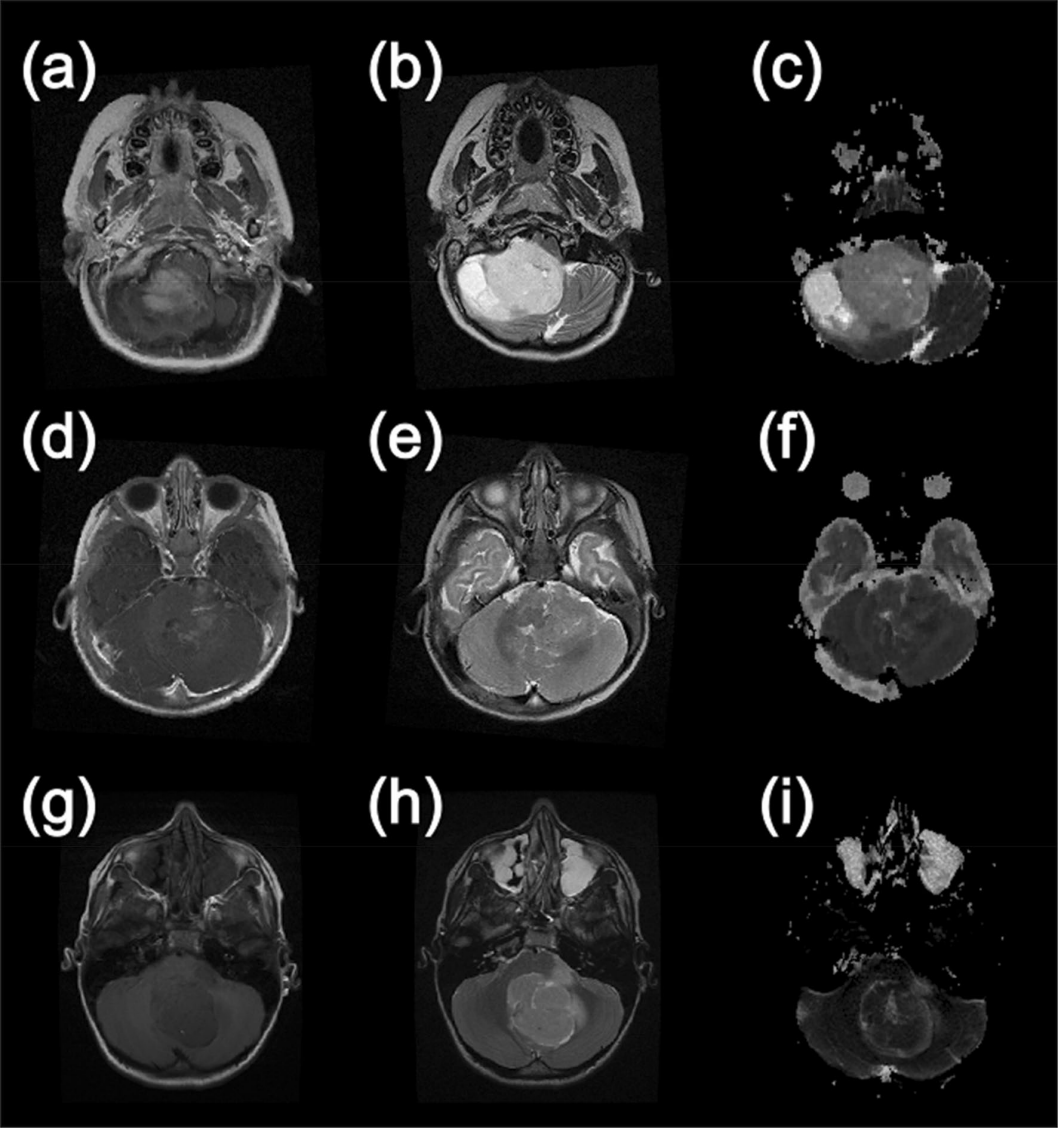
Example MR images from paediatric brain tumour patients. This first column shows T1-weighted images following the injection of gadolinium contrast agent. The second column shows T2-weighted images and the final column shows apparent diffusion coefficient maps calculated from diffusion-weighted images. (**a**–**c**) are taken from a patient with a Pilocytic Astrocytoma, (**d**–**f**) are from a patient with an Ependymoma and (**g**–**i**) were acquired from a patient with a Medulloblastoma.

Example MRIs are shown for the most common types of posterior fossa paediatric brain tumours in Fig. 1. As can be seen in the images, the ADC maps look distinctly different for the Medulloblastomas and the Pilocytic Astrocytomas with higher ADC observed for the latter. The appearance of the Ependymomas and the Medulloblastomas are much closer in terms of ADC contrast.

## Comparison of mean ADC values

**Figure 2:**
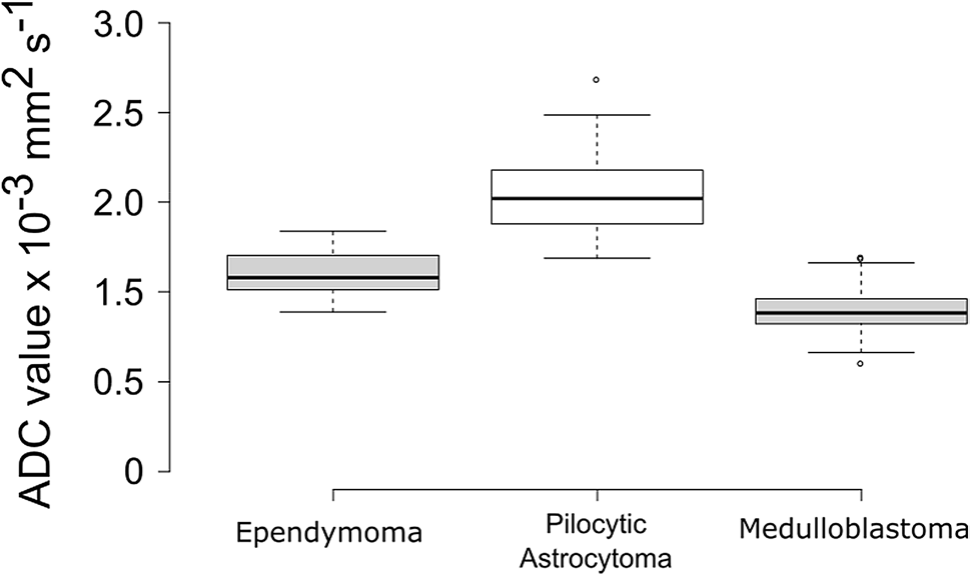
A boxplot showing the distribution of the means from the ADC histograms for Ependymomas, Pilocytic Astrocytomas and Medulloblastomas. Centre lines show the medians; box limits indicate the 25th and 75th percentiles as determined by R software (R Core Team (2017). R: A language and environment for statistical computing. R Foundation for Statistical Computing, Vienna, Austria. URL https://www.R-project.org/) whiskers extend 1.5 times the interquartile range from the 25th and 75th percentiles, outliers are represented by dots.

The mean values for the tumours were as follows: Ependymoma (EP) 1.126 ± 0.155 × 10^−3^ mm^2^ s^−1^, Medulloblastoma (MB) 0.870 ± 0.015 × 10^−3^ mm^2^ s^−1^, Pilocytic Astrocytoma (PA) 1.656 ± 0.029 × 10^−3^ mm^2^ s^−1^. An ANOVA of the group means showed significant differences between the PAs and the EPs and the MBs and the EP (*P* < 0.001). The box plots in Fig. 2 show overlap spread of the mean ADC values of the PA and EP and also between the MB and EPs. A receive operator curve was constructed for MB and EP to determine an optimal cut off value for the mean values for the two tumour types. The cut off value was found to be 0.984 × 10^−3^ mm^2^ s^−1^ with a sensitivity of 80.8% and a specificity of 80.0% with 21/26 EP and 48/55 MB falling either side of the cut off boundary.

**Figure 3:**
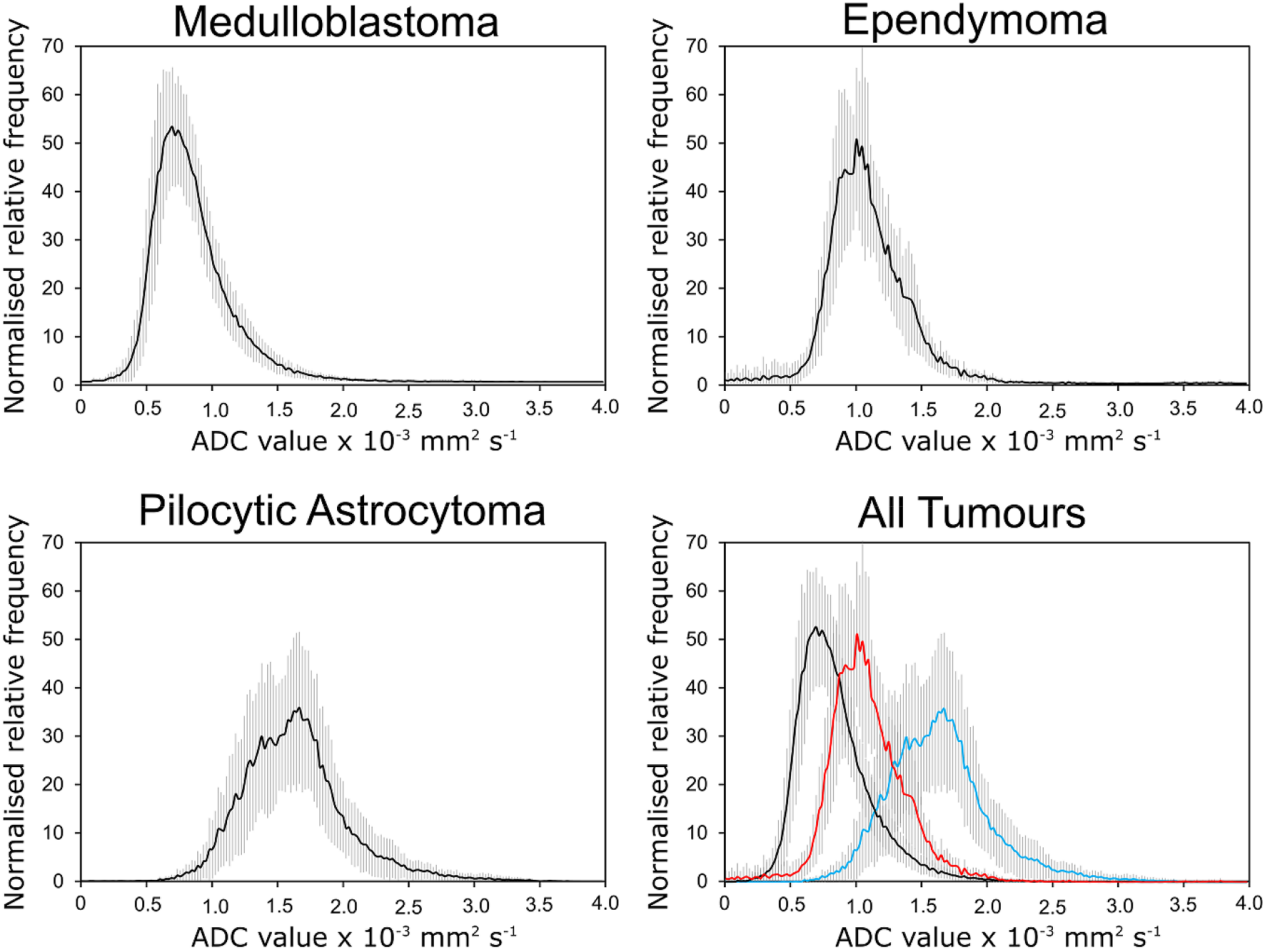
Mean normalised histograms for the most common paediatric posterior fossa brain tumours. The shaded areas show the standard deviation. The bottom right shows histograms for the different tumour types overlaid with the black representing Medulloblastomas, red representing Ependymoma sand blue representing Pilocytic Astocytomas.

Mean histograms from the three main tumour types are presented in Fig. 3 including the standard deviations. Also included is a plot illustrating the overlap of the mean histograms.

## Posterior fossa tumour classification

Pilocytic Astrocytomas, Medulloblastomas and Ependymomas were classified using the extracted parameters (mean, variance, skew, kurtosis and 10th and 20th etc.). Two classification methods were employed, Naïve Bayes (NB) and Random Forest (RF). NB was chosen as the more simple model and RF the more complex for comparison purposes (Table 2).

**Table 2:** The classification results using both Naïve Bayes (NB) and Random Forest (RF).

	Predicted group membership						Percentage correct		Total correct	
	Ependymoma		Medulloblastoma		Pilocytic astrocytoma					
	NB	RF	NB	RF	NB	RF	NB	RF	NB	RF
Observed										
Ependymoma	21	19	2	2	3	5	80.8	73.1	21/26	19/26
Medulloblastoma	9	3	46	52	0	0	83.6	94.5	46/55	52/55
Pilocytic Astrocytoma	9	3	0	0	32	33	88.9	91.7	32/36	30/36
Overall							84.6	86.3	99/117	101/122

Using the extracted histogram parameters, as shown in Table 2, the overall classification accuracy was 84.6% using NB and 86.3% using RF. The balanced overall accuracy using the same data was 84.4% for NB and 86.3% for RF. The largest discrepancies were observed for Ependymomas where NB classified 80.8% of cases correctly and RF classified 73.1% correctly. The opposite trend was observed for Medulloblastomas where NB classified 83.6% correctly and RF classified 94.5% correctly.

**Figure 4:**
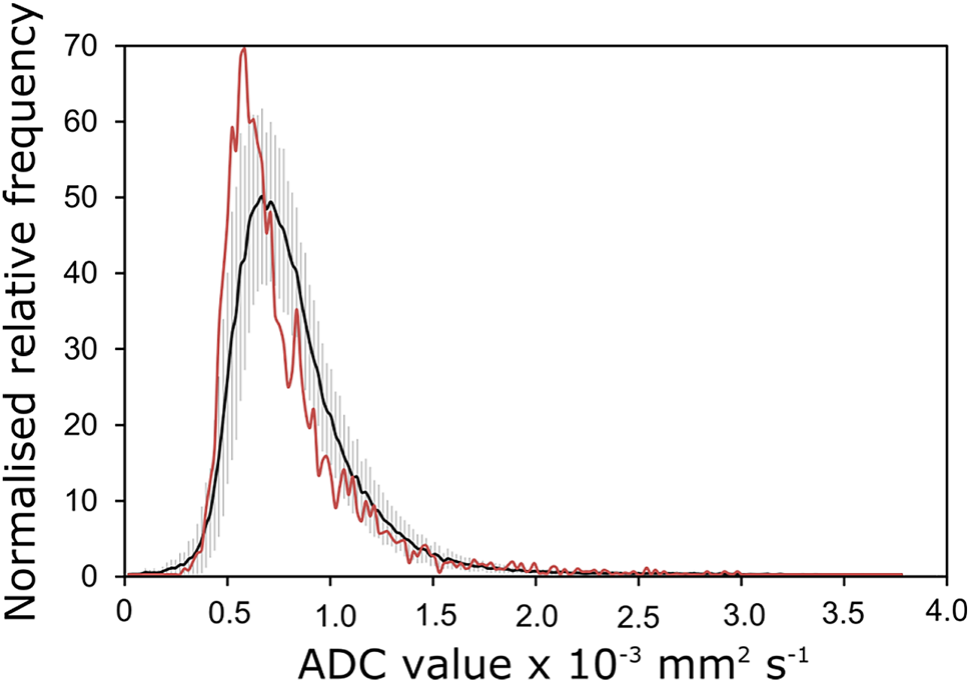
A plot showing the average histograms for both the Medulloblastomas, n = 55 (black) and the ATRTs, n = 4 (red).

A plot showing a comparison between the average histograms of four ATRTs and the medulloblastomas used for the classification is shown in Fig. 4. The overlap between the ATRTs and the medulloblastomas in terms of the histograms is clear from the plot.

Histograms of the rare low grade tumours are presented in the supplementary material.

## Discussion

One of the most simplistic image analyses that can be performed on parametric maps is the mean values from a region of interest drawn around or within a lesion. This approach is potentially available on most clinical PACS systems and would provide a feasible route for quantitative measures of DWI to be integrated into routine clinical practice. We found that there was a significant difference between the mean tumour values when observing Pilocytic Astrocytomas, Medulloblastomas and Ependymomas *P* < 0.001. These differences are in line with previous single centre studies that have shown significant differences between Medulloblastomas and Ependymomas^8,14,15^ which are traditionally the two tumour types which overlap in terms of ADC. It is encouraging that a large multicentre study can show similar statistical differences to single centre studies despite the heterogeneity in acquisition protocol and scanner model and manufacturers. We used an ROC analysis which suggested a cut off value between the Medulloblastomas and Ependymomas of 0.984 × 10^−3^ mm^2^ s^−1^ with high sensitivity (80%) and specificity (80.8%). We believe this may be the best route for the use of ADC clinically in the most common scenario of discriminating between Medulloblastomas and Ependymomas as it can be achieved on most clinical PACS systems. This will however, need to be prospectively validated.

The average histograms presented in Fig. 2 show that there are differences between the three main tumour types if assessed as groups and they have distinct appearances. The Medulloblastomas in general have the peaks at the lowest ADC values followed by the Ependymomas and then the Pilocytic Astrocytomas. However, the standard deviation represented by the error bars, suggests there is the potential for significant overlap on a case-by-case basis. While these plots may be helpful to clinicians as part of a larger diagnostic workup they have limited use on a case-by-case basis without additional information. Interestingly, the Medulloblastomas appear to be the most homogeneous tumours with respect to their histograms. The Pilocytic Astrocytoma ADC distribution has increased variance with respect to the shape of the average histogram which is in keeping with their histological appearance which is heterogeneous and micro cystic. This suggests the measurement of more than one microenvironment.

Classification of the tumours using the histogramswas performed using metrics extracted from the histogram such as skew, variance and quantiles, as had been used in previous studies^4,8^. We also used two different classifiers with both linear (Naïve Bayes) and non-linear (Random Forest) approaches to see if the classification was significantly affected. As differentiation of the Ependymomas from the other two tumour types has been the most challenging it is appropriate that this classification test takes precedence when selecting the best classifier. A Random Forest classifier used on the extracted histogram parameters resulted the best overall classification accuracy of 86.3% but the highest classification rates for Ependymomas of 80.8% were obtained using a Naïve Bayes classifier.

Overall the classification rates are not as high as has previously been seen in the literature for ADC analysis with Rodriguez Gutierrez et al.^8^ quoting overall classification rates of 91.4% and Bull et al. 93.75%. However, our study is much larger than the aforementioned studies with a more heterogeneous data input with regards to hospitals, scanners and acquisition protocols.

Historically analysis of ADC images has been qualitative with areas of lowest signal being used for identification of cellular density^18,19^. This has also propagated into quantitative measurements where minimum ADC is used as a biomarker for diagnosis and also treatment monitoring. This approach can be challenging from a data analysis perspective and although it can seem attractive and time efficient does result in a large amount of data being discarded. When the whole region of interest is preserved and histogram analysis is used the data is far less sensitive to user error^20^, especially if the regions of interest are larger. If this approach is also combined with feature selection and machine learning algorithms, then large datasets can be used to better effect.

As is shown in Fig. 4 ADC does have its limitations when attempting to distinguish between ATRTs and Medulloblastomas. The average histograms display a large amount of overlap and when the ATRTs were run through the classifier were all classified as Medulloblastomas. The rare tumour types presented in the supplementary material were included for illustrative purposes and also illustrate the difficulty that these cases present when attempting to classify.

The results from this study underline the potential opportunity for ADC maps to be used in multicentre studies. We have shown that a cohort of patients scanned on a wide range of different scanners, in different hospitals and using heterogeneous protocols can still produce reliable results. This has potential for integration of advanced imaging techniques into clinical trials for the assessment of treatment efficacy. Our results suggest that strict harmonisation of DWI protocols may not be necessary for the production of reliable biomarkers thereby allowing the utilisation of historically acquired imaging data as demonstrated here. This is important as harmonisation of protocols is challenging across multiple centres, especially for routinely acquired images.

## Study limitations

This is a retrospective study of previously acquired data with the histological diagnosis already known. The classifiers and cut off values from this study will therefore need to be tested on prospective data to be truly validated. Likewise, the machine learning classifiers should also be tested prospectively. ADC is unable to discriminate ATRTs from Medulloblastomas as the diffusion parameters were virtually identical. The study has been limited mainly to the three most common types of brain tumours in the posterior fossa and did not address the identification of rarer tumour types such as choroid plexus papillomas. No in-depth analysis has been conducted of the tumour genetic or molecular subtypes, due to limitations of statistical power. The regions of interest were hand drawn so were susceptible to human error, although this issue was addressed through the use of multiple experts to verify the regions of interest which were shown to be reproducible across raters. The cut off values used in the study were produced from ADC maps from bespoke software, care must therefore be used when implementing this in ADC maps produced directly by scanner software or third-party vendors. Despite this, it is likely that the results would be applicable to ADC maps produced by scanners since the calculation of ADC maps involves a linear fit between two data points which is inherently stable, although this should be formally validated. Care would need to be taken when DWI is acquired with multiple b-values to ensure that ADC values were calculated from the b0 and b1000 images in order to make the results compatible with those of this study. The histogram metrics were produced via bespoke software which creates a barrier to clinical implementation, however we envisage these metrics becoming increasingly available via scanner manufacturers and clinical picture archiving and communication systems (PACS).

## Conclusions

We have shown in this study that it is possible to discriminate between the three most common types of paediatric posterior fossa brain tumour types using histogram analysis of Apparent Diffusion Coefficient maps in a large cohort of patients acquired on a multi-centre basis.

## Supplementary Information

Supplementary Information.
