# Supplementary material for: Classification of paediatric brain tumours by diffusion weighted imaging and machine learning
Source: Sci Rep. 2021 Feb 4;11:2987. doi: 10.1038/s41598-021-82214-3 (PMC7862387; doi:10.1038/s41598-021-82214-3)
Supplement: Supplementary file 1 — Supplementary Information. [file 41598_2021_82214_MOESM1_ESM.docx]

Classification of Paediatric Brain Tumours by Diffusion Weighted Imaging and Machine Learning

Jan Novak^1,2,3,4^, Niloufar Zarinabad^1,2^, Heather Rose^1,2^, Theodoros Arvanitis^1,2,5^, Lesley MacPherson^6^, Benjamin Pinkey^6^, Adam Oates^6^, , Patrick Hales^7^, Richard Grundy^8^, Dorothee Auer^9,10^, Daniel Rodriguez Gutierrez^8,11^, Tim Jaspan^8,12^, Shivaram Avula^13^, Laurence Abernethy^13^, Ramneek Kaur^7^, Darren Hargrave^14^, Dipayan Mitra^15^, Simon Bailey^16^, Nigel Davies^1,2,17^, Christopher Clark^7^, *Andrew Peet^1,2^

1. Institute of Cancer and Genomic Sciences, School of Medical and Dental Sciences, University of Birmingham, Birmingham, UK.

2.Oncology, Birmingham Women’s and Children’s NHS foundation trust, Birmingham, United Kingdom.

3.Department of Psychology, School of Life and Health sciences, Aston University, Birmingham, United Kingdom.

4. Aston Neuroscience Institute,School of Life and Health sciences, Aston University, Birmingham, United Kingdom.

5. Institute of Digital Healthcare, WMG, University of Warwick, Coventry, United Kingdom.

6. Radiology, Birmingham Women’s and Children’s NHS foundation trust, Birmingham, United Kingdom.

7. Developmental Imaging & Biophysics Section, UCL Great Ormond Street Institute of Child Health, London WC1N 1EH, United Kingdom

8. The Children’s Brain Tumour Research Centre, University of Nottingham, Nottingham, United Kingdom.

9. Sir Peter Mansfield Imaging Centre, University of Nottingham Biomedical Research Centre, Nottingham, United Kingdom.

10. NIHR Nottingham Biomedical Research Centre, Nottingham, United Kingdom

11. Medical Physics, Nottingham University Hospital, Queen’s Medical Centre, Nottingham, United Kingdom

12. Neuroradiology, Nottingham University Hospital, Queen’s Medical Centre, Nottingham, United Kingdom

13. Department of Radiology, Alder Hey Children’s Hospital NHS Foundation Trust, Liverpool, United Kingdom

14. Haematology and Oncology Department, Great Ormond Street Children's Hospital, London, United Kingdom

15. The Newcastle upon Tyne Hospitals NHS Foundation Trust, Newcastle, United Kingdom

16. Sir James Spence Institute of Child Health, Royal Victoria Infirmary, Newcastle upon Tyne, United Kingdom

17. Radiation Protection Services, University Hospitals Birmingham NHS Foundation Trust, Birmingham, United Kingdom

Corresponding Author Details

Professor Andrew Peet

Institute of Cancer and Genomic Sciences,

School of Medical and Dental Sciences,

University of Birmingham,

Birmingham,

UK.

[a.peet@bham.ac.uk](mailto:a.peet@bham.ac.uk)

Supplementary material

Supplementary Figure 1: A summary of the diffusion-weighted imaging protocols used at the sites


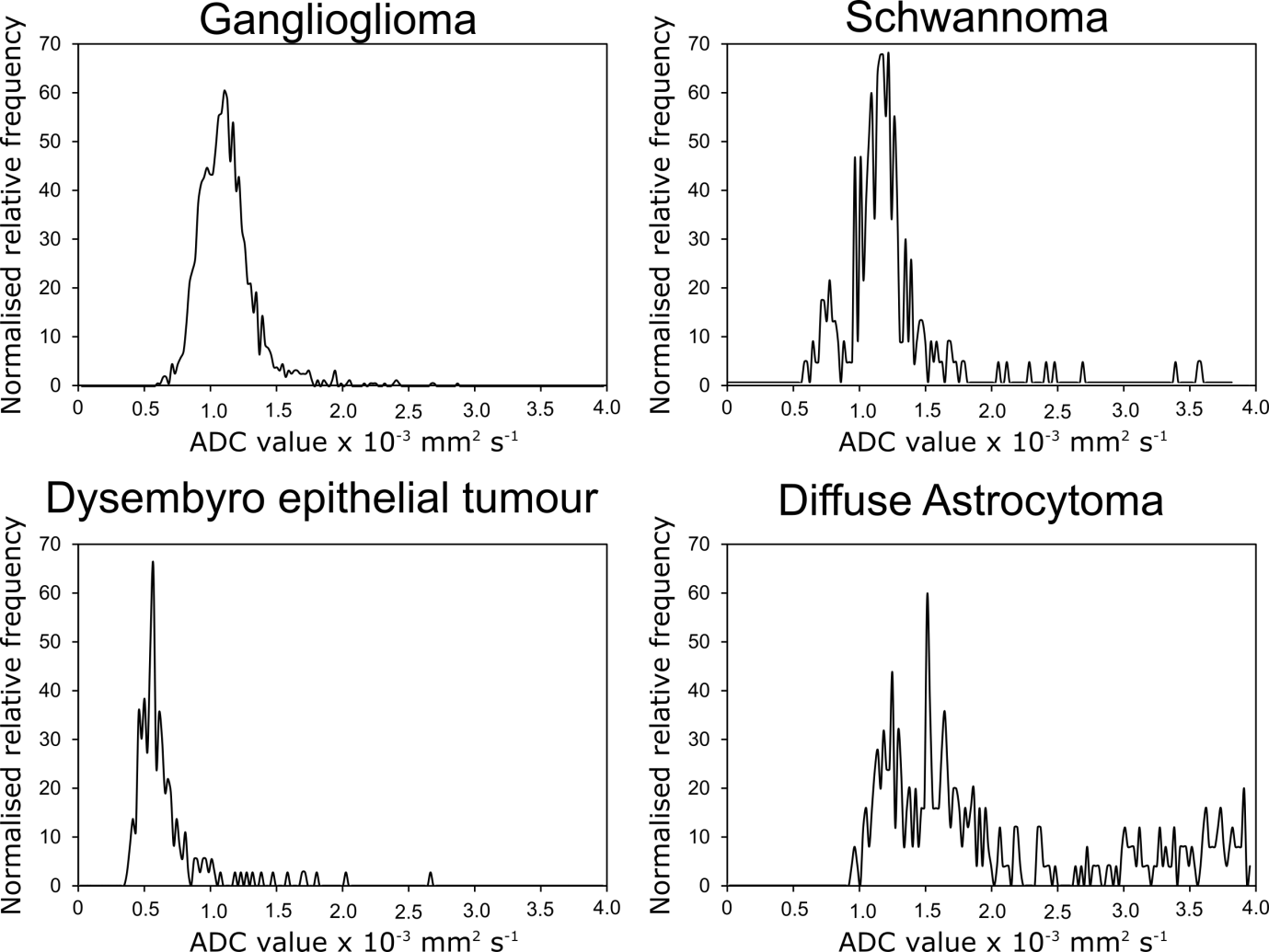


Supplementary Figure 2: A figure showing ADC histograms of some of the rare tumours found in pediatric patients. The following tumour types were included in the plot; Ganglioglioma (n = 2), Schwannoma (n = 1)


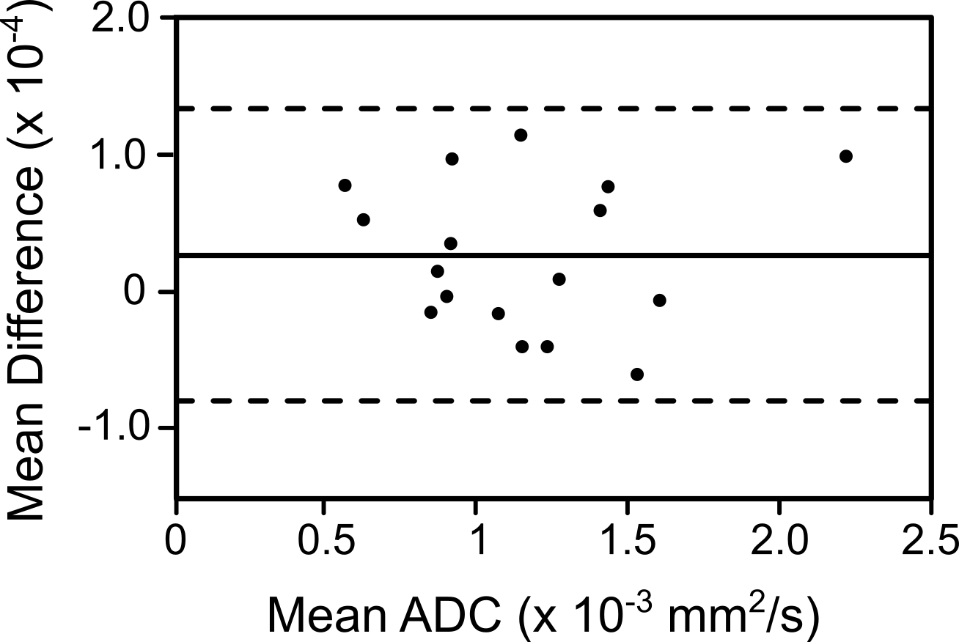


Supplementary Figure 3: A Bland-Altman plot showing the agreement for mean ADC values between different raters that delineated tumor regions of interest.
